# Households' perception of climate change and human health risks: A community perspective

**DOI:** 10.1186/1476-069X-11-1

**Published:** 2012-01-11

**Authors:** Md Aminul Haque, Shelby Suzanne Yamamoto, Ahmad Azam Malik, Rainer Sauerborn

**Affiliations:** 1 Institute of Public Health, Heidelberg University, Im Neuenheimer Feld 324, 69120 Heidelberg, Germany; 2 Department of Population Sciences, University of Dhaka, Dhaka-1000, Bangladesh; 3 Federal Postgraduate Medical Institute, Lahore, Lahore-54600, Pakistan

**Keywords:** Bangladesh, households' perception, climate variability, health risks, climate-induced health problems

## Abstract

**Background:**

Bangladesh has been identified as one of the most vulnerable countries in the world concerning the adverse effects of climate change (CC). However, little is known about the perception of CC from the community, which is important for developing adaptation strategies.

**Methods:**

The study was a cross-sectional survey of respondents from two villages--one from the northern part and the other from the southern part of Bangladesh. A total of 450 households were selected randomly through multistage sampling completed a semi-structure questionnaire. This was supplemented with 12 focus group discussions (FGDs) and 15 key informant interviews (KIIs).

**Results:**

Over 95 percent of the respondents reported that the heat during the summers had increased and 80.2 percent reported that rainfall had decreased, compared to their previous experiences. Approximately 65 percent reported that winters were warmer than in previous years but they still experienced very erratic and severe cold during the winter for about 5-7 days, which restricted their activities with very destructive effect on agricultural production, everyday life and the health of people. FGDs and KIIs also reported that overall winters were warmer. Eighty point two percent, 72.5 percent and 54.7 percent survey respondents perceived that the frequency of water, heat and cold related diseases/health problems, respectively, had increased compared to five to ten years ago. FGDs and KIIs respondents were also reported the same.

**Conclusions:**

Respondents had clear perceptions about changes in heat, cold and rainfall that had occurred over the last five to ten years. Local perceptions of climate variability (CV) included increased heat, overall warmer winters, reduced rainfall and fewer floods. The effects of CV were mostly negative in terms of means of living, human health, agriculture and overall livelihoods. Most local perceptions on CV are consistent with the evidence regarding the vulnerability of Bangladesh to CC. Such findings can be used to formulate appropriate sector programs and interventions. The systematic collection of such information will allow scientists, researchers and policy makers to design and implement appropriate adaptation strategies for CC in countries that are especially vulnerable.

## Background

Weather and climate affect the key determinants of human health: air, food and water. They also influence the frequency of heat waves, floods and storms as well as the transmission of infectious diseases [1,2]. The global climate is altering dangerously due to various natural and anthropogenic reasons such as increasing fossil fuel combustion and industrial and agricultural activities, which emit carbon dioxide and greenhouse gases [3]. Worldwide, climate change-related impacts including prolonged flooding, heat waves, drought, sea level rises, salinity, temperature and rainfall variations have become evident [4]. People are directly exposed to changing weather patterns (temperature, precipitation, sea-level rises and more frequent extreme events) and indirectly through changes in the quality of water, air and food in addition to changes in ecosystems, agriculture, industry, human settlements and the economy. These forms of direct and indirect exposure can lead to death, disability and suffering [5,6]. The World Health Organization (WHO) has estimated that globally over 150,000 deaths annually result from changes in the world's climate, relative to the average from the baseline climate of 1961-1990 [7,8]. The Fourth Assessment Report (AR4) of the Intergovernmental Panel on Climate Change (IPCC) clearly states that climate change is contributing to the global burden of disease and premature deaths [9]. Thus, there is a growing need for a better understanding of the multi-faceted and complex linkages between global environmental change and human health as well as the establishment of an international research community to address such issue [10,11].

Both developed and developing countries are facing the adverse effects of climate change, such as prolonged floods and severe drought in South Asia and Africa, heat waves in Europe, devastating cyclones and tidal surges (e.g. Hurricane Katrina and Rita) along the Atlantic coasts [4]. Compared to developed countries, those that are still developing are more vulnerable to climate change and climate variability [11]. Climate change is projected to increase threats to human health, particularly in lower income populations and tropical/subtropical countries [2,12]. These effects are compounded by poor socioeconomic conditions and weak health systems. Among the many developing countries, Bangladesh is also highly vulnerable to climate change [4,6]. According to the Global Climate Risk Index 2009 of *GermanWatch*, Bangladesh is the most vulnerable country in the world [13]. It has already experienced various climate change-related events such as heat waves, cold waves, flood, drought, salinity intrusion and cyclones in recent years that have caused direct and indirect adverse impacts on human health [14]. The impact of climate change on individuals will vary, according to many factors like age, social class, occupation and gender [15,16]. Vulnerable groups like the poor and, in particular, poor women will be affected most by the probable impacts of climate change [17]. Women in Bangladesh are generally the poorest of the poor [18] and therefore are expected to be the most vulnerable to the effects of climate change in this region. Further, a high population density, low level of literacy, low per capita income, high level of poverty, subsistence focus, resource poor setting, inadequate infrastructure and long coastal belt have made the climate vulnerability of the country more severe [6].

In Bangladesh, the association between climate variability and hospital visits for non-cholera diarrhea has been studied [19]. Matsuda and De Magny also studied the effect of climate change on the disease of cholera [20]. Furthermore, the probable direct and indirect impacts of climate change on health have also been studied using secondary data [6]. In fact, most of the studies, except one by the Climate Change Cell (CCC) of Bangladesh [11] and the World Bank [21], were based on the use of secondary data. The CCC not only identified climate change and health as a priority issue but also highlighted the possible overall impacts of climate change on health [11,22]. Likewise, Rahman also emphasized the need to study the health effects of climate change as well as develop responses and possible actions that could be taken to reduce the health impacts of climate change in Bangladesh [5].

Nonetheless, little research to date has been conducted in Bangladesh concerning the perceptions of households and their coping strategies regarding the effect of climate change on their health. Perceptions on climate change and health have already been studied in developing [23,24] and developed countries [25-28]. These studies have been conducted among students, educators, farmers and scientists and have shown that there should be an emphasis on the understanding of local or community responses to climate. However, what still missing is research on climate change perceptions and its effect on human health conducted at the community level. As communities are vulnerable to climate change, their perceptions of climate variability can be used in the development of national adaptation program of actions (NAPAs), climate change strategy papers or sector programs. For example, our respondents mentioned that rainfall was a problem, which strongly suggests that policy makers need to consider irrigation as an intervention in the future data including people's perceptions of climate change and its effect on climate sensitive diseases and health problems are necessary in order to formulate and implement any kind of response or intervention regarding climate vulnerability. The objective of this study was to explore households' perceptions of climate change (changes to heat, cold and rainfall). This study also explored people's knowledge of the effects of climate change on diseases and other health problems in their region.

## Methods

Perceptions on climate/climate variability were assessed using a mixed methods research design [29-31]. Data collection was conducted between September 2010 to March 2011 in two villages; one from the Rajshahi district in the northern part of Bangladesh and the other from the Khulna district in the south. We purposely selected districts from two different climatic zones to obtain a wider range of household perceptions. The northern part of Bangladesh has been categorized as a heat/drought prone area whereas the southern part is defined as a flood-vulnerable area [11]. However, the socio-economic, educational, occupation, demographic and agricultural pattern of the two areas are similar [32,33]. Multistage sampling was used to select two villages for this observational study. We have selected one Upozila (third level government administrative unit) from each district, one union (local level government administrative unit) from each Upozila and one village from each union randomly. Oral or written consent was obtained from each participant. Ethical approval was obtained from the Ethical Commission of Heidelberg University, Germany. The study was also approved by the research evaluation committee of the Department of Population Sciences, University of Dhaka, Bangladesh.

Data were collected using both quantitative and qualitative instruments. To ensure the validity and reliability of the instruments, we flagged the issues related climate variability in the literature and then consulted with experts while developing each of the items related to heat, cold and rainfall in the questionnaire and interview guide. We also compared the items with those included in other studies. Lastly, we pre-tested and field-tested the instruments, and revised them to produce the final questionnaire and interview guide. The surveys were administered by both female and male interviewers who were involved in the process of developing the data collection tool, which facilitated their understanding of the concepts and questions. All interviewers also received training on rapport building, confidentiality and social and cultural sensitivity during data collection. A senior researcher was present in the field full time to monitor and ensure the quality of the data collected.

For the quantitative part, the survey was administered to 450 (238 male and 212 female participants) randomly selected households of the two villages. The response rate of the survey respondents was 97.82 percent and no respondent dropped out after starting the interview. The national sex ratio was used as reference in selecting the number of male and female respondents for the study [32]. The survey was administered to one person within the randomly selected households. Among males, the household head or eldest member was interviewed. The eldest male and female members were selected for the interviews for the purpose of soliciting perceptions of climate variability changes over time and its effect on agriculture, health, diseases and overall livelihoods. Among females, the eldest member of the household was also interviewed. Lists of all the households (total 2250) in the two villages were prepared and 450 households were selected from the list for interview using probability proportionate sampling.

The survey was a semi-structured questionnaire that assessed demographic characteristics, perceptions of heat, cold and precipitation, perceived effects of heat, cold and rainfall on agriculture, farming and everyday life as well as perceived links to human health problems/diseases. Respondent were asked to report their perceptions of changes in heat, cold and precipitation relative to those events five to ten years ago. There were three sections in the questionnaire. Socio-demographic information of the respondent and their family members was documented at the beginning of the interview. Section 'A' included 45 questions regarding perceptions about heat. Section 'B' included 33 questions on perceptions about cold. Section 'C' included 14 questions regarding perceptions about precipitation, rainfall and flooding. In each section, respondents were also asked to report on the health problems/diseases most frequently associated with that event(s). Background information of the respondents were given in the Table 1. Responses in the Tables 2 and 3 were designed to be reported on a Likert scale ("very low", "low", "normal", "high", "very high"). Rest of the solicited responses in the Tables 4, 5, 6, 7 and 8 were categorical ("Yes", "No", "Don't know" and "Not Applicable"). Quantitative data were analyzed using Statistical Package for the Social Sciences (Version SPSS-12.0 and SPSS-17.0).

**Table 1:** Characteristics of the survey participants (n = 450)

Household Characteristics of Respondents	Total 450	%
**Sex (per cent)**		

Male	238	52.9

Female	212	47.1

Sex ratio (male: female)	1: 0.89	

**Age, mean (SD)yr**		

Overall	39.9	

Male	42	

Female	35	

**Education**		

No Formal Education	121	26.9

Primary Education	112	24.9

Junior Secondary	94	20.9

Secondary School Certificate (SSC)	49	10.9

Higher Secondary (HSC)	36	8.0

Bachelor Level	25	5.6

Masters level	13	2.9

Total	450	100.0

**Occupation**		

Agricultural activities	140	31.1

Homemaker/Housewife	187	41.6

Services (Govt. NGO,)	48	10.7

Business (small and medium)	54	12.0

Others (village doctor, rickshaw puller, unemployed, fisherman)	21	4.7

Total	450	100.0

**Household Information**		

Family income (monthly mean of lower 50% in BDT*)	4438.00	

Family income (median in BDT*)	7000.00	

Range of family income (BDT*)	1,000.00-35.000.00	

Mean family size (persons)	4.15	

Range of family size (persons)	2-7	

* US$1 = BDT 74.00 in 2011

**Table 2:** Perceptions of heat, cold and rainfall changes compared to five to ten years ago (N = 450, male 238, female 212)

Climate Perceptions		Sex				Total	
				
		Male		Female			
		
		Frequency	%	Frequency	%	Frequency	%
What is the present intensity of heat during summer in comparison to the last five to ten years? ***Χ^2^. p < 0.000; N = 450***	Very low	1	0.2	0	0.0	1	0.2
	
	Low	9	2.0	4	0.9	13	2.9
	
	Normal	4	0.9	2	0.4	6	1.3
	
	High	163	36.2	193	42.9	356	79.1
	
	Very high	61	13.6	13	2.9	74	16.4
	
	**Total**	**238**	**52.9**	**212**	**47.1**	**450**	**100**

What is the present bitterness of cold during winter in comparison to the last five to ten years? ***Χ^2^. p < 0.000; N = 450***	Very low	5	1.1	3	0.7	8	1.9
	
	Low	151	33.6	135	30.0	286	63.6
	
	Normal	39	8.7	9	2.0	48	10.7
	
	High	38	8.7	65	14.2	103	22.9
	
	Very high	5	1.1	0	0.0	5	1.1
	
	**Total**	**238**	**52.9**	**212**	**47.1**	**450**	**100**

What is the present intensity of rainfall during rainy session in comparison to the last five to ten years? ***Χ^2^. p < 0.000; N = 450***	Very low	73	16.2	16	3.5	89	19.7
	
	Low	87	19.3	185	41.1	272	60.4
	
	Normal	9	2.0	8	1.8	17	3.8
	
	High	53	11.8	3	0.7	56	12.5
	
	Very high	16	3.6	0	0.0	16	3.6
	
	**Total**	**238**	**52.9**	**212**	**47.1**	**450**	**100**

**Table 3:** Perceptions of changes in frequency of disease occurrence attributable to climate variability by sex (N = 450, male 238, female 212)

Climate Change and Disease Perceptions		Sex				Total	
				
		Male		Female			
		
		Frequency	%	Frequency	%	Frequency	%
What is your overall perception about diseases due to climate variability in your locality? ***Χ^2^. p < 0.280; N = 450***	Increased	196	43.6	165	36.1	362	80.2
	
	Decreased	26	5.8	34	7.6	60	13.3
	
	No change	16	3.6	13	2.9	28	6.2
	
	**Total**	**238**	**52.9**	**212**	**47.1**	**450**	**100**

What is the present frequency of diseases during summer compared to five to ten years earlier? ***Χ^2^. p < 0.036; N = 450***	Very Low	1	0.2	0	0.0	1	0.2
	
	Low	25	5.6	36	8.0	61	13.6
	
	Normal	17	3.8	11	2.4	28	6.2
	
	High	184	40.9	163	76.9	347	77.1
	
	Very high	11	2.4	2	0.4	13	2.9
	
	Total	**238**	**52.9**	**212**	**47.1**	**450**	**100**

What is the present frequency of disease during winter in comparison to five to ten years ago? ***Χ^2^. p < 0.005; N = 450***	Very low	4	0.9	1	0.2	5	1.1
	
	Low	31	6.9	54	12.0	85	18.9
	
	Normal	19	4.2	10	2.2	29	6.4
	
	High	180	40.0	146	32.4	226	72.4
	
	Very high	4	0.9	1	0.2	5	1.1
	
	**Total**	**238**	**52.9**	**212**	**47.1**	**450**	**100**

What is the present frequency of disease during rainy season in comparison to five to ten years ago? ***Χ^2^. p < 0.000; N = 450***	Very low	23	5.1	1	0.2	24	5.3
	
	Low	31	6.9	99	22.1	130	29.0
	
	Normal	34	7.6	10	2.2	43	9.6
	
	High	144	32.0	102	22.8	245	55.0
	
	Very high	6	1.3	0	0.0	6	1.3
	
	**Total**	**238**	**52.9**	**212**	**47.1**	**450**	**100**

**Table 4:** Perceived changes in heat, cold and rainfall compare to five to ten years ago (n = 450)

Perceived changes in Climate Variability	Heat		Cold		Rainfall	
	
	Yes	No	Yes	No	Yes	No
**What were the changes respondents perceived due to heat over time?**	%	%				

Has the intensity of heat increased during summer?	95.3	4.7				

Are heat waves more frequent now during summer/drought?	77.3	22.7				

Are there more frequent and strong storms during summer/drought?	40.3	59.7				

Is there enough rainfall during summer/drought?	33.8	76.2				

Do hailstorms occur more frequently?	16.0	84.0				

Do the canals and lakes carry enough water during summer/drought?	2.0	98.0				

Do the rivers carry enough water during summer/drought?	7.3	92.7				

Can you cultivate your crops regularly using rain water during summer?	22.9	77.1				

Are you restricted from being outside of your work due to extreme heat?	18.4	81.6				

Can you fetch drinking water from tube-well?	58.9	37.6				

Does the deep tube-well reach the water table?	47.3	52.7				

Does the deep tube-well maintain the same water pressure throughout summer?	37.3	62.7				

Has any deep tube-well run out of water during summer/drought?	46.4	28.9				

**What were the changes they perceived due to winter/cold?**			%	%		

Is the severity of cold more irregular than in the past?			72.4	27.6		

Does winter arrive on time?			17.1	82.9		

Does the winter season remain for a longer period of time?			14.0	86.0		

Is the frequency of rain the same as previously?			25.3	74.7		

Do cold waves occur with the same frequency as in the past?			83.5	16.5		

Has the intensity of fog decreased compared to previous days?			73.6	26.4		

Is there the presence of dew drops along with fog?			85.1	14.9		

Does the severity of cold linger for a shorter period of time?			96.7	3.3		

Does the dense fog conceal sunlight for more than a day?			61.1	38.9		

Is the timing of the sunrise delayed?			88.0	12.0		

**What were the changes they perceived due to rainfall?**					%	%

Is the occurrence of rainfall the same as previously?					1.3	98.7

Do rainfall events occur for 5-7 days during the rainy season?					1.3	98.7

Does rainfall occur regularly during rainy season?					1.1	98.9

Are the ponds full during rainy season?					6.7	92.9

Does flooding occur during rainy season?					3.6	96.2

Can you cultivate your crops using rain water?					3.6	76.7

Does rainfall occur on time?					0.9	99.1

Does rainfall occur irregularly?					67.1	32.9

Has the frequency of lightning increased?					4.8	81.4

Percentage of "don't know" not presented in the table						

[Respondents were asked the question: "What were the changes you perceived due to heat, cold and rainfall over time"?]

**Table 5:** Perceived problems due to extreme heat and cold (n = 450)

Perceived problems due to extreme climate variability	Heat		Cold		Consequences of the problems
		
	Yes	No	Yes	No	
**Perceived problems due to heat**	%	%			

Problems with drinking water	70.7	29.3			Health and hygiene

Cannot cultivate the crops in due time	75.8	4.7			Production loss

Growth of crops has decreased	78.2	2.2			Production loss

Yield of crops has decreased	77.1	3.1			Production loss

Cannot go outside of house due to extreme heat	87.3	9.3			Working hour loss

Have to work hard for irrigation	80.8	1.1			Extra work

Diseases/health problems/sickness has increased	96.2	3.1			Health problem

**Perceived problems due to extreme cold**			**%**	**%**	

Boro (summer paddy) cannot be cultivated timely			68.9	3.1	Food shortage

Boro (summer paddy) seedbed cannot be sown			70.9	0.9	Production loss, food shortage

Potato cultivation is hampered			16.2	49.3	Food production loss

Betel leaf field is hampered			63.6	1.6	Cash crop loss

Robi (winter crops) crops cannot be cultivated			47.1	26.2	Cash crop loss

Potato cultivation is hampered because of dense fog			60.7	2.7	Cash crop loss

Flowering/blooming is delayed			47.1	16.5	Cash and Food loss

Color of the crops has faded			45.1	17.5	Cash loss

Mango inflorescence is hampered due to heavy fog			46.0	16.8	Cash and Food loss

[Respondents were asked the question: "What are the problems you face because of changes in heat and cold"?]

Percentage of "don't know" has not been presented in the table

**Table 6:** Perceived diseases/health problems due to changes in heat, cold and rainfall/flood, compared to five to ten years ago (N = 450).

Types of diseases	During Heat (*yes %)	During Cold (*yes %)	During Rainfall (*yes %)
Normal cold/cough/fever	85.8	96.7	92.4

Dysentery	66.5	55.9	49.4

Headache	65.0	64.0	55.9

Diarrhoea	63.3	25.2	56.8

Skin diseases	41.2	49.3	42.4

Burning sensation	35.7	5.3	7.3

Conjunctivitis	23.0	6.8	7.7

Jaundice/Hepatitis B	22.6	2.7	5.8

High blood pressure	19.2	5.6	7.3

Skin burn/blistering	18.9	0.7	3.6

Asthma	16.2	40.6	18.0

Psychological disorders	15.1	3.9	6.7

Typhoid	14.0	2.7	6.7

Pox	10.8	1.9	3.9

Weight loss	7.6	10.6	4.6

Malnutrition related diseases	6.2	6.5	5.8

Rheumatism/aching	4.9	13.8	8.0

Pneumonia	4.6	25.5	8.5

Measles	4.1	1.2	3.6

Bleeding from nose	3.8	0.2	3.9

Heatstroke	3.5	0.5	4.1

Malaria	2.2	0.5	8.2

Dengue	0.8	0.5	4.6

[Respondents were asked the questions:

"Which diseases/health problems occur most frequently in your area due to changes in heat compared to five to ten years ago"?

"Which diseases/health problems occur most frequently in your area due to changes in cold/winter compared to five to ten years ago"?

"Which diseases/health problems occur most frequently in your area due to changes in rainfall compared to five to ten years ago"? ]

*Only the percentage of 'Yes' has been presented in the table

**Table 7:** Perceived future and current major hazards or disasters due to climate change (N = 450)

Perceived future and current major hazards or disasters	Problem currently facing respondents (*Yes %)	Problem can occur in future (*Yes %)
Drought	95.3	97.3

Flood	6.6	14.8

Storm	15.2	25.2

Cyclone	26.1	30.3

Rise in sea level (Tidal surge)	12.5	19.4

Salinity	30.4	34.0

Water logging	5.1	4.6

[Respondents were asked the questions: "What are the major natural disasters you have experienced due to climate variability"? "What are the major natural disasters you predict will occur from climate variability"?]

*Only percentage of "Yes" has been only presented in the table

**Table 8:** Sources of perceive changes in heat, cold and rainfall, compared to five to ten years ago (N = 450)

Sources of perceived changes	During Heat		During Cold	
	
	Yes	No	Yes	No
**Sources of perceived changes in heat**	%	%		

Heat wave/feel	99.3	0.7		

Cannot go outside of house due to extreme heat	98.0	2.0		

Increased sweating	99.8	0.2		

Burning sensation/blistering	73.7	26.3		

Feel tired	70.4	29.6		

Cannot sleep at night due to extreme heat and sweating	70.9	29.1		

Tin roof of the house becomes too hot	66.5	33.5		

Lack of rainfall for a long period	99.8	0.2		

Tender leaves of trees die	97.6	2.4		

Lower levels of water in ponds, canals, river [34]	99.1	0.9		

Layer of deep tube wells has decreased	92.4	7.6		

Land has become much drier and now cracks	99.3	0.7		

Irrigation water dries up very soon in the field	92.6	7.4		

Feel more thirsty	72.5	27.5		

More frequent dry storms	71.1	28.9		

Growth of the trees has decreased	66.4	33.6		

TV news	72.5	27.5		

Radio news	17.0	87.0		

Newspaper	18/1	81.9		

**Sources of perceived changes in cold**			%	%

TV news			64.9	32.0

Radio news			10.4	85.6

Newspaper			15.1	81.3

Cold waves have increased			100.0	0.0

People's experience			98.0	1.8

Dense fog (increased)			98.9	.9

Dew drops like rain form more frequently			97.8	2.0

No sun until 10.30 a.m due to strong fog			97.3	2.4

Limited visibility of sun for few days			93.3	6.5

[Respondents were asked the questions: "What are the sources you use to determine whether the level of heat has changed?" "What are the sources you use to determine whether the level of cold has changed?]

Percentage of don't know has not been presented in the table. Modern technologies or techniques were not available to determine exact changes in heat, cold and rainfall levels.

For qualitative methods, twelve focus group discussions (FGDs) (six with females and six with males) and 15 key informant interviews (KIIs) were conducted by the research team using an interview guideline on the broad themes of heat, cold and rainfall. Interviews were recorded with the permission of the participants and played back to the respondents. FGD and KII included the same issues and items on climate variability which were used in the survey. Around ten to twelve participants attended every FGD. The participants were selected purposively. FGDs and KIIs participants were senior community members, non-governmental organization officials, village doctors, local political leaders and teachers of a socio-demographic background similar to that of the survey participants from the study areas. All FGDs and KIIs were transcribed and analyzed according to themes (e.g. heat, cold, rainfall and health problems).

## Results

A total of 53 per cent of the survey respondents were male and 47 per cent were female (Table 1). The mean age of males was 42 years and 35 years for females. About 27 per cent had no formal education and 24.9 per cent had only primary education. A third (31.1 per cent) of the respondents was farmers and 41.6 per cent of the respondents were homemakers. The mean household income of the lower 50 per cent of the respondents was BDT. 4438.00/month and the median income was BDT 7000.00/month *(US$1 = BDT 74.00 in 2011) *for a household of 4.15 persons.

Most of the survey respondents perceived that changes in climate variability had occurred compared to five or ten years ago. There were no significant differences between males and females in terms of their perceptions regarding climate variability. Over 95.5 per cent of the respondents reported that the heat during summers had increased and 80.2 per cent reported that rainfall had drastically decreased, compared to five to ten years ago (Table 2). The majority (63.5 per cent) also reported that it was not as cold during the winters. FGD and KII participants also reported that summers were hotter, winters were warmer and that there was less rain during the rainy seasons. However, they also reported experiencing very erratic and severe cold for a period of 5-7 days each winter, which temporarily restricted their activities. They also affirmed that the cold weather had a very destructive effect on agricultural production, their everyday life and health, especially that of children and the elderly. FGD and KII respondents identified climate change as a serious problem for their daily life, crop cultivation, health and overall livelihoods.

Survey respondents were also asked to report their overall perceptions of the frequency of climate variability-induced diseases and health problems in their areas. They perceived that the frequency of climate variability-induced diseases and health problems had increased because of changes in heat, cold and rainfall events, compared to five to ten years ago (Table 3). Eighty per cent and 72 per cent perceived that health problems related to heat and cold, respectively, had also increased. FGD and KII members also reported a rise in climate variability-induced diseases in their areas. They also remarked that treatment facilities had improved but that the number of patients and diseases had also correspondingly increased. For example, a male farmer (FGD participant), from Rajshahi district mentioned that "nowadays new and unknown diseases and outbreaks are often identified among the members of the community".

We also asked survey respondents to give their views on different parameters related to changes in heat, cold and rainfall (Table 4). Included were 13 aspects related to heat, 10 aspects related to cold and nine aspects related to rainfall changes. The response percentages from the survey respondents for each parameter reveal the perceived changes in climate variability and its related aspects compared to five to ten years ago. More than seven types of immediate problems from changes in heat and cold were mentioned (Table 5): health and hygiene, production loss, working hour losses, extra work, poor crop growth, poor crop yield, over irrigation and increased illness incidence. Reoccurring fever/cough/cold, dysentery, headaches, diarrhea, skin diseases, burning sensation, conjunctivitis, jaundice, blisters, asthma, pox, weight loss and pneumonia were the health issues the most frequently reported in relation to extreme and irregular patterns of heat, cold and rainfall (Table 6).

In addition to the issues mentioned in Tables 5 and 6, survey respondents also predicted future environmental problems or hazards they perceived would occur from climate variability (Table 7). Respondents reported that more droughts (97.3 per cent), storms (25.2 per cent), cyclones (30.3 per cent) and salinity issues (34.0 per cent) might occur in the future. Interestingly, in a flood prone country, only 14.8 per cent of the survey respondents predicted that floods might occur in the future.

More than 20 different sources or indications were used by the survey participants to identify perceived changes in heat and cold (Table 8). All the sources, except radio, TV and newspapers, were largely associated with environmental observations (e.g. crop yields) within their locality. Equipment such as thermometers or gauges was not available in respondents' assessments of changes in heat, cold or precipitation.

## Discussion

The majority of the survey respondents were rural people with little formal education whose livelihood is mainly based on agriculture. Nevertheless, they have clear perception of the changes in heat, cold and rainfall that have occurred and its effects on their livelihood and health. According to the majority of respondents, climate variability is perceived to have changed and resulted in an increase in climate variability sensitive diseases, human health issues and agricultural problems [34,35]. They reported that rainfall has dropped, heat has increased and the longevity of winter has decreased over time. Overall, winters were reported as warmer but erratic, irregular and bitter cold was still experienced for short periods (5-7 days). They have already experienced catastrophic natural disasters and also predicted more natural disasters in the future due to climate variability. All respondents' perceptions of climate variability are largely drawn from proxy indicators like production losses, poor growth of crops, increased sickness as well as the shared experiences of other members of the community. No modern techniques and technologies were available to inform community members about weather forecasts in advance. Instead, members become sick, lose their crops and suffer other losses, which they then attribute to extreme heat or cold events or unprecedented floods or storms. We assume that the participants in this study were not familiar with research on climate variability but their perceptions echo the findings [4,6,12] about changes in heat, cold and rainfall [36] reported in the literature.

To our knowledge, the present study is among the first to assess households' perceptions of climate variability in Bangladesh. Other studies regarding perceptions of climate change have been conducted in India, Nepal [36], the USA [27,28,37], Canada, Malta [38,39], Ethiopia [36] and Australia [40]. Respondents in these studies were given a set of factors related to climate change to choose from and the results presented descriptively. In our study, we also used the same approach but added a qualitative aspect to assess the reliability and validity of our quantitative findings. The inclusion of the perceptions of both males and females has also added a new dimension to the existing literature as there is less information regarding gender in relation to climate change, especially in developing countries [16,17]. These CV-related issues have been validated among a large number of male and female respondents and the reliability of the tools checked in comparisons with those from other studies in other countries [21,27,28,36,38]. The CV aspects we have used to determine people's perceptions of climate change and its effect on health problems can also be useful for other researchers.

In developed countries perception studies have been conducted mainly to explore mitigation options [27,28,37-40]. In developing countries, perception studies have been conducted in relation to adaptation options [36] Participants of these studies perceived that the climate had changed and that these changes resulted in a wide range of negative effects on health, agriculture and livelihood. In our study we also aimed to obtain in-depth information from affected people to be used in the development of effective adaptation measures (e.g. NAPA, specific sector programs, climate change adaptation strategies and action plans) for climate vulnerable people, with special focus on the health sector. These findings address an important knowledge gap and provide significant information to policy makers.

Researchers, national and international experts and governments use scientific literature to determine the vulnerability of Bangladesh to climate change [4,6,11,21]. We assume that the community members of this study are not familiar with the scientific literature regarding climate change yet provide an essential perspective on the issue can be useful for policy makers. A better understanding of local knowledge and coping strategies are needed for the successful formulation of adaptation or mitigation measures [41-44]. In the development of the National Adaptation Program of Actions (NAPAs) in 2008 for climate change for Bangladesh, the government heavily depended on information from stakeholder meetings, mathematical models and reports [42]. No field studies were available to provide community information for the NAPAs. Thus, communities' understanding and predictions of changes in climate are an important source of information for policy makers looking to develop and implement effective and sustainable adaptation measures for Bangladesh, as well as other countries.

The Bangladesh Climate Change Strategy and Action Plan (BCCSAP) presents a 10-year plan to build the capacity and resilience of Bangladesh to meet the challenges of a changing climate. The plan envisions a financial need of about $5 billion during the first five years through 2014 [45,46]. To combat climate change impacts, the Government of Bangladesh established a climate change fund with a total annual allocation of approximately US$100 million per year starting in 2009-2010 [47]. However, major challenges facing the BCCSAP are the identification of vulnerable sectors, the prioritization of action and the effective and efficient use of funds [48]. Such study findings can help policy makers to select the most vulnerable sectors and prioritize the action needed for the immediate benefit of communities. In addition, based on the information on the frequency of diseases, sickness and health problems, specific and effective health sector programs can be formulated or revised and community level health systems improved for the climate vulnerable [44]. Furthermore, the fourth pillar of the BCCSAP is to use "research and knowledge management to predict the likely scale and timing of climate change impacts on different sectors of the economy and socio-economic groups; to underpin future investment strategies; and to ensure that Bangladesh is networked into the latest global thinking on climate change" [41]. These research findings will also contribute to the process of achieving the fourth pillar of the BCCSAP.

We have surveyed a large number of households in the study but only within two villages, so the findings may be more applicable to an in-depth understanding of these issues. The generalization of these perceptions to other parts of Bangladesh may require further research. Additionally, the issue under study is complicated and difficult to measure. Thus, we had to use different proxy indicators instead of direct measures. Additionally, there might be recall bias, as we also had to depend on the subjective judgments of the respondents' previous experiences.

## Conclusions

Study participants had clear perceptions about the changes in heat, cold and rainfall that had occurred over the last five to ten years. Local perceptions of changes in climate variability included increased heat, overall warmer winters, reduced rainfall and fewer floods. They also perceived that the effects of the changes in climate variability were mostly negative on means of living, human health, agriculture and overall livelihoods. Community members described how every aspect of their life was affected by the changing and erratic pattern of heat, cold and rainfall. Increased sickness and health problems due to climate variability were specifically mentioned. Most local perceptions on climate variability were consistent with the scientific evidence regarding the vulnerability of Bangladesh to climate change. Participants also linked climate variability to current problems and identified important future threats to themselves, families and livelihoods. Based on these findings, appropriate health sector programs and interventions can already be formulated. The systematic collection of such information will allow scientists, researchers and policy makers to design and implement appropriate adaptation strategies for climate change in countries that are especially vulnerable.

## List of Abbreviations

CC: Climate Change; FGD: Focus Group Discussion; KII: Key Informant Interview; CV: Climate Variablity; WHO: World Health Organization; AR4: The fourth Assesment Report; IPCC: Intergovernmental Panel for Climate Change; CCC; Climate Change Cell; NAPA: National Adaptation Program of Actions; BCCSAP: Bangladesh Climate Change Strategy and Action Plan (BCCSAP); USD: United State Dollar; UNFPA: United Nations Population Fund; EMMA: Erasmus Mundas Mobility in Asia.

## Competing interests

The authors declare that they have no competing interests.

## Authors' contributions

MAH designed the study, developed the questionnaire, supervised the data collection, analyzed the data and wrote the paper. SSY contributed to the interpretation of the findings as well as the drafting and writing of the manuscript. AAM contributed to the questionnaire study design and analysis of the data. RS contributed to the development of the overall study concept, design of the study and drafting of the paper. All authors read and approved the final manuscript.
